# Bloom syndrome with lung involvement

**DOI:** 10.4103/0970-2113.53234

**Published:** 2009

**Authors:** Girija Nair, Ivona Lobo, T. K. Jayalaksmi, Abhay Uppe, Savita Jindal, Abhishek Chandra, Shivani Swami

**Affiliations:** *Department of Tuberculosis and Chest, Dr. D. Y. Patil Medical College, India*

**Keywords:** Bloom's syndrome, telangiectasia, growth retardation, respiratory infections, consanguinous parentage

## Abstract

We report a case of a 24-year old male presented with cough and breathlessness with diabetes mellitus and diagnosed as a case of bloom syndrome. He was a product of consanguineous marriage, having short stature, dolicocephaly, polydactyly, prominent nose with telangiectasia face. The respiratory system examination revealed bilateral coarse crepitations and wheezes and the chest X-ray revealed emphysema with right middle zone inhomogenous opacity. Also, CT thorax examination revealed bilateral cystic bronchiectasis with bronchiolitis obliterans. Bloom's syndrome was diagnosed on the basis of clinical features.

## INTRODUCTION

Bloom syndrome is a rare autosomal recessive disorder in which patients have typical facial features and short stature. They are prone to recurrent respiratory infections and diabetes mellitus.

## CASE REPORT

A 24-year male patient presented with cough and breathlessness since childhood, even more since five months. A product of consanguinous marriage among first cousins, he has history of growth retardation and delayed milestones with no history of mental retardation. He was diagnosed to have diabetes mellitus (DM) before a month and was on human insulin subcutaneously.

### On general examination

**Figure 1:**
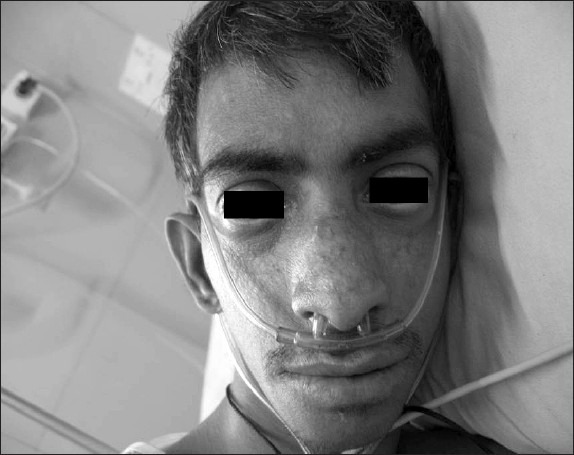
Telangiectasia over cheeks and nose and prominent nose, dolicocephaly

He had short stature, height 117 cms, dolicocephaly, small face, bilateral malar telangiectasia, prominent nose, polydactyly were seen [Figure 1]. Secondary, sexual characters were present, absence of eyelashes bilaterally (madarosis), clubbing-Grade II present, cyanosis and tachypnoea with respiratory rate 35/min were present. Respiratory system revealed bilateral coarse crepitations and occasional wheeze.

Cardiovascular system and abdomen were normal.

On CNS examination, he was conscious, well oriented to time, place, and person.

Haematological investigations revealed normocytic, normochromic anaemia with thrombocytopenia; FBS-397 mg/dl and PLBS-377 mg/dl on long acting subcutaneous insulin. Thyroid function tests, liver function tests, renal function tests, and serum electrolytes, within normal limits. ABG was suggestive of respiratory acidosis. Chest X-ray (PAView) showed emphysema with bilateral upperzone inhomogenous opacity.

CT thorax revealed bilateral cystic bronchiectasis with emphysema with patchy ground glass attenuation in anterior segment of both upper lobes suggestive of bronchiolitis obliterans with right apical fibrosis with pleural thickening.

CT brain showed calcification in bilateral globi pallidi and right cerebellar hemisphere and subtle hypodensities in bilateral periventricular white matter.

The serum FSH (22.73 mIU/ml) was slightly increased. USG abdomen was normal, 2 D echo was normal. Serum leutinising hormone and serum prolactin were within normal limit.

Genetic studies including sister chromatid exchange (SCE) levels were attempted on two separate occasions, but no cell growth could be obtained.

## MANAGEMENT

Our patient was treated as a case of type 2 respiratory failure with insulin dependent diabetes mellitus (IDDM) in ICU with antibiotics, bronchodilators, corticosteroids, insulin, and low flow oxygen. The condition of the patient improved, after which he was shifted to the ward. Patient was discharged in fair general condition after 2 months of hospitalization, on long-term oxygen therapy (LTOT).

Patient came back to casualty after one month with severe dyspnoea and died of cardio-respiratory arrest.

## DISCUSSION

### Disease name/synonyms

*Bloom-torre-mackacek syndrome (BS)/Congenital telangiectatic erythema:* BS is a rare human autosomal recessive disorder characterized by marked genetic instability associated with a greatly increased predisposition to a wide range of cancers. The predominant clinical features are constant pre and postnatal growth retardation, dolicocephaly, narrow facies with nasal prominence and malar and mandibular hypoplasia, facial sun sensitive telangiectatic erythema in butterfly areas, café au lait spots, recurrent respiratory and gastrointestinal infections and DM.[1]

BS was first described in 1954, occurs in approximately 1 in 48,000 in ashkenazic jewish population, it is rare in the general population.[2,3]

Bloom syndrome belongs to a chromosomal breakage syndrome, typically autosomal recessive in transmission. The hallmark is an approximate 10 fold increase in the rate of SCEs compared to normal cells; this is the only objective criterion for diagnosis.

## ETIOLOGY

Bloom syndrome arises through mutations in both copies of BLM gene which is located on chromosome 15 at 15q 26.1 frameshift or nonsense mutations which lead to premature termination codon and missense mutations have been found in BLM gene from BS patients.

## CLINICAL FINDINGS

Bloom syndrome registry exists in New York in which 168 BS - 93 males and 75 females were reported till 1991.

The average height was described as: 130-162 cm (for males), 122-151 cm (for females).

The other features are:

1. Bird like facies with narrow face and prominent nose and malar, mandibular hypoplasia.
2. Sun sensitive telangiectatic erythema over butterfly area of face.
3. Café au lait spots.
4. High pitched voice.
5. Repeated gastrointestinal infections during infancy.
6. DM at a mean age of 24 years.
7. In males small testes with total failure of spermatogenesis and early cessation of menstrual cycle in females.
8. Recurrent respiratory and gastrointestinal infections due to immunodeficiency.
9. Minor anatomical abnormalities such as obstructing anomalies of urethra.
10. Average intelligence or mental deficiency.
11. Congenital thrombocytopenia, mild anaemia, asthma.[4]

### Laboratory diagnosis

SCE detection based on differential labelling of sister Chromatids.

Screening for BLM gene mutations by analysis of the 21 coding exons.

Antenatal diagnosis: SCE analysis of fetal cells, identifying specific BLM gene mutation.

Our patient presented with short stature, telangiectasia face and DM. He was the product of a consanguineous marriage among first cousins. This muslim patient did not give any history of having a Jewish or Arabic ancestry. The CT thorax reports revealed emphysema, bronchiectasis and areas of bronchiolitis obliterans. The presence of telengiectasia leads to the search for other causes such as ataxia telangiectasia due to mutation of gene.

There was no mental retardation, he had studied upto 8^th^ standard.

We diagnosed him as probably bloom's syndrome (BS) since he had many of the features of this syndrome. The lung lesions of bronciectasis, broncholitis obliterans and emphysema could be due to repeated respiratory infections in childhood. People having BS are known to have repeated respiratory and gastrointestinal infections due to immunodeficiency.

The confirmation of diagnosis is by:

1. A 10-fold increase in SCEs compared to normal cells.
2. Screening for BLM gene mutations could also be performed by the analysis of the 21 coding exons and it is possible to look for specific changes in BLM gene.

We attempted to look for increased SCEs by culture of blood lymphocytes on two separate occasions but there was no cell growth; thus we could not confirm our diagnosis.

### Differential diagnosis

Fanconi anaemias and xeroderma pigmentosa are chromosomal breakage syndromes related to BLM proteins.

Ataxia telangiectasia is a chromosomal breakage syndrome, usually associated with thymic hypoplasia and variable T cell deficiency. There are decreased levels of S. Ig G2 and Ig A with normal Ig M. Cerebellar ataxia is seen by three years of age with ocular and cutaneous telangiectasia and chronic sinopulmonary disease. These patients have a strong tendency to develop lymphomas. Other primary immunodeficiency diseases are CVIDs (common variable immunodeficiency diseases). There are recurrent pneumonia, empyema, bronchiectasis, hemolytic anaemias, rheumatoid arthritis, and also gastro intestinal diseases.

Short limb dwarfism is usually associated with T and B cell immune defects.

The management of BS patients is usually symptomatic. Since there is no curative treatment for BS, the physicians should carefully follow BS patients to ensure early diagnosis of cancer.

Genetic counseling is to be offered to the parents as there is an autosomal recessive transmission mode in BS; siblings of heterozygous carriers are at 25% risk of having BS and 50% risk of being a carrier.

We are presenting this case of a short statured man having bronchiectasis and brochiolitis obliterans with DM and facial telangiectasia, as probably BS. Most of the features fit into this syndrome, which is autosomal recessive in transmission; in this patient who is a product of consanguineous marriage among first cousins. BS is very rare in the general population, the frequency being 1 in 48,000 for ashkenazic jewish population.

## References

[1] German J (1993). Bloom Syndrome: A Mendelian prototype of somatic mutational disease. Medicine.

[2] Bloom D (1954). Congenital telengiectatic erythema resembling lupus erythematosus in dwarfs. Am J Dis Child.

[3] Shahrabani-Gargir L, Shomrat R, Yaron Y, Orr-Urtreger A, Groden J, Legum C (2003). High frequency of a common bloom syndrome DNA helicase. BMC Cell Biol.

[4] German J (1995). Bloom's syndrome. Dermatol Clin.

